# In Vivo Measurement of Cervical Elasticity on Pregnant Women by Torsional Wave Technique: A Preliminary Study

**DOI:** 10.3390/s19153249

**Published:** 2019-07-24

**Authors:** Paloma Massó, Antonio Callejas, Juan Melchor, Francisca S. Molina, Guillermo Rus

**Affiliations:** 1 Instituto de Investigación Biosanitaria, ibs.GRANADA, 18012 Granada, Spain; 2 San Cecilio University Hospital, 18016 Granada, Spain; 3 Department of Structural Mechanics, University of Granada, 18071 Granada, Spain; 4 Excellence Research Unit, “Modelling Nature” (MNat), University of Granada, 18071 Granada, Spain

**Keywords:** torsional wave, cervix, pregnancy, cervical stiffness

## Abstract

A torsional wave (TW) sensor prototype was employed to quantify stiffness of the cervix in pregnant women. A cross-sectional study in a total of 18 women between 16 weeks and 35 weeks + 5 days of gestation was performed. The potential of TW technique to assess cervical ripening was evaluated by the measurement of stiffness related to gestational age and cervical length. Statistically significant correlations were found between cervical stiffness and gestational age ($R^{2}=0.370$, p=0.0074, using 1 kHz waves and $R^{2}=0.445$, p=0.0250, using 1.5 kHz waves). A uniform decrease in stiffness of the cervical tissue was confirmed to happen during the complete gestation. There was no significant correlation between stiffness and cervical length. A stronger association between gestational age and cervical stiffness was found compared to gestational age and cervical length correlation. As a conclusion, TW technique is a feasible approach to objectively quantify the decrease of cervical stiffness related to gestational age. Further research is required to evaluate the application of TW technique in obstetric evaluations, such as prediction of preterm delivery and labor induction failure.

## 1. Introduction

Approximately, 15 million babies are born preterm (before 37 weeks of gestation) per year, i.e., more than 1 in 10 newborns, and this number is rising in both developing countries and Europe [1,2]. Worldwide, complications of preterm birth are the main cause of child mortality under five years of age [1,3]. Prematurity often leads to long-term disabilities such as learning, visual and hearing problems. Clinical and social risk factors of preterm birth have been identified to develop feasible and cost-effective care measures to save children [1]. Nonetheless, a high proportion of spontaneous preterm birth remains unpredictable.

Current models based on cervical length, obstetric history, digital vaginal examination and echography of the cervix are not able to accurately predict a preterm birth with sufficient anticipation, and there is a lack of evidence on how to prevent preterm delivery [4,5]. Even though there is an agreement that cervical ripening plays a fundamental role during pregnancy, histological changes and biomechanical properties of the cervix are not entirely characterized. The current lack of a clinical tool for the quantitative evaluation of the biomechanic parameters of the cervix is probably a barrier to advance in preventing spontaneous preterm birth [6]. Since 2012, the WHO is encouraging to accelerate research into the causality of preterm birth, and to test effective approaches that would lead to save babies. Recently, elastography techniques are being put forward in the literature to assess quantitatively the stiffness of the cervix as a promising tool to estimate preterm birth risk, as well as to predict the success of labor induction [7,8,9,10,11,12].

Quasi-static elastography methods have been used to evaluate the cervical stiffness at different gestational ages. The result is a qualitative deformation gradient map, called elastogram. However, all methods have shown unclear results regarding reproducibility and associations between stiffness and gestational age [13,14,15,16]. The measurements are also liable to the sensor pressure applied by the clinician, which is not accounted for.

In contrast, dynamic elastography techniques have the strength to provide the absolute quantitative values of stiffness as an objective criterion to evaluate the process of cervical ripening [17,18]. This technology relies on shear ultrasonic waves that travel through the soft tissue. The measurement of the shear wave propagation speed allows characterizing shear stiffness. The commercially available dynamic Supersonic Shear Imaging (SSI) technique employs ultrasonics radiation force to generate shear waves. Shear wave speed in cervix was statistically significant lower in women delivered preterm and inpatients with preterm uterine dynamics compared to women delivered at term [12]. Peralta et al. [19] evaluated SSI elastography to quantify cervical stiffness in real time and its evolution in induced labor in ewes, concluding that stiffness decreases during maturation in induced labor. The Acoustic Radiation Force Imaging (ARFI) is based on displacements generated by an ultrasound beam using the same imaging probe. ARFI has already been proved to evaluate differences in mature versus immature cervical tissue ex vivo [20] and in vivo in pre- and post-labor induction [21]. Both studies agree that shear waves speeds are statistically significantly different in mature versus immature cervical tissue. Viscoelasticity maps of uterine corpus and cervix were assessed thought magnetic resonance elatography in nonpregnant women [22]. Results show a higher elasticity in uterine corpus, and similar viscosity compared with cervix.

The presented measurements data are taken using an alternative dynamic technique: torsional wave (TW) technique [23]. This is based on the propagation of shear waves through the tissue not only in depth but also radially, which makes the technique suitable for applications such as cervical tissue. Axis-symmetric waves allows the precise interrogation of soft tissue mechanical functionality in cylindrical geometries, which are challenged by current elastography approaches in small organs.

This work was aimed at evaluating the reliability and feasibility of TW technique to provide consistent data on the changes of the cervical stiffness during pregnancy. Eighteen singleton-pregnant women were recruited. The hypothesis were: (1) torsional wave technique has the capacity to quantify cervical stiffness defined by its elastic modulus; and (2) stiffness decreases along pregnancy. The second hypothesis stems from the fact that the cervical tissue behavior depends on changes in its multi-scale structure from a mechanical point of view. The cervical stroma microstructure is formed of cross-linked mesh of collagen immersed into viscous proteoglycan [24]. These biochemical compounds exist on different scales whose length is variable by several orders of magnitude. They provide a tractive and compressive strength to the cervical tissue. Despite the fact that cervical architecture changes during ripening [25], these modifications along pregnancy are still not well studied.

## 2. Materials and Methods

### 2.1. Design of the Study

A cross-sectional study in healthy pregnant women was performed to assess stiffness modifications in cervix.

### 2.2. Healthcare Settings

The pre-pilot test study was carried out at San Cecilio University Hospital in Granada. The data were analyzed in the Ultrasonics Laboratory in the University of Granada.

### 2.3. Ethical Issues

The study met the principles of the Declaration of Helsinki. Approvals of the Ethical Committee in Human Research of University of Granada and Ethical Commission and Health Research of San Cecilio University Hospital in Granada were achieved.

### 2.4. Subjects

Eighteen healthy women were recruited from their routine medical visits during pregnancy, and TW technique explorations were performed in the Fetal Medicine Unit. The entire population of women in the study had pregnancies without any complication with a median of 26.4 (16 weeks to 35 weeks + 5 days) gestation weeks, and there was no twin pregnancy. A statistical power analysis was designed to estimate the size of the population. A multivariate continuous regression with a power of 80%, estimated significance in a two-tail distribution, and a recommended effect size ES = 0.30, yielded a sample size of 17 subjects. Exclusion criteria were multiple pregnancies, previous cervical surgeries and patients with information relative to malignant changes in the cervical tissue. All women enrolled in the evaluation provided agreement by signing a written consent and reading the information of the patient report.

**Figure 1:**
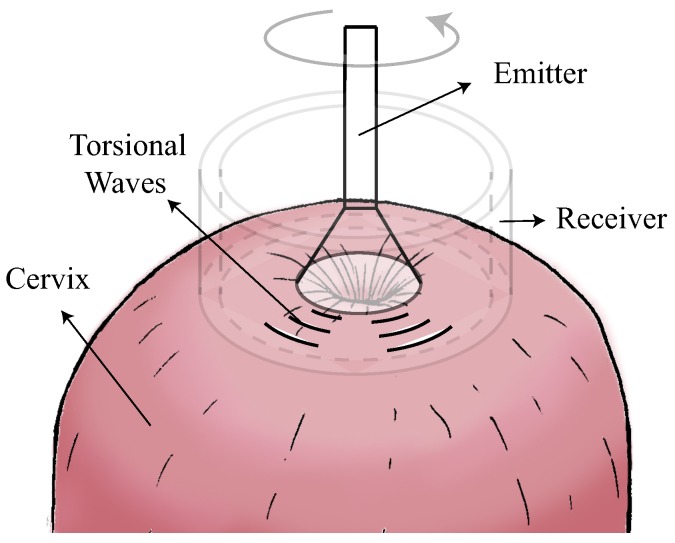
Schematic diagram for the exploration with TW technique.

For the exploration with TW technique, the participants emptied their bladder before the exploration and then were placed in the dorsal lithotomy position. The intravaginal device was allocated in contact with the cervical internal OS (see Figure 1). The measurements of cervical length were obtained by a transvaginal sonography probe, which was directed in the anterior fornix. A sagital view was obtained. Three TW technique and cervical length measurements per women were performed.

### 2.5. Torsional Wave Technique

**Figure 2:**
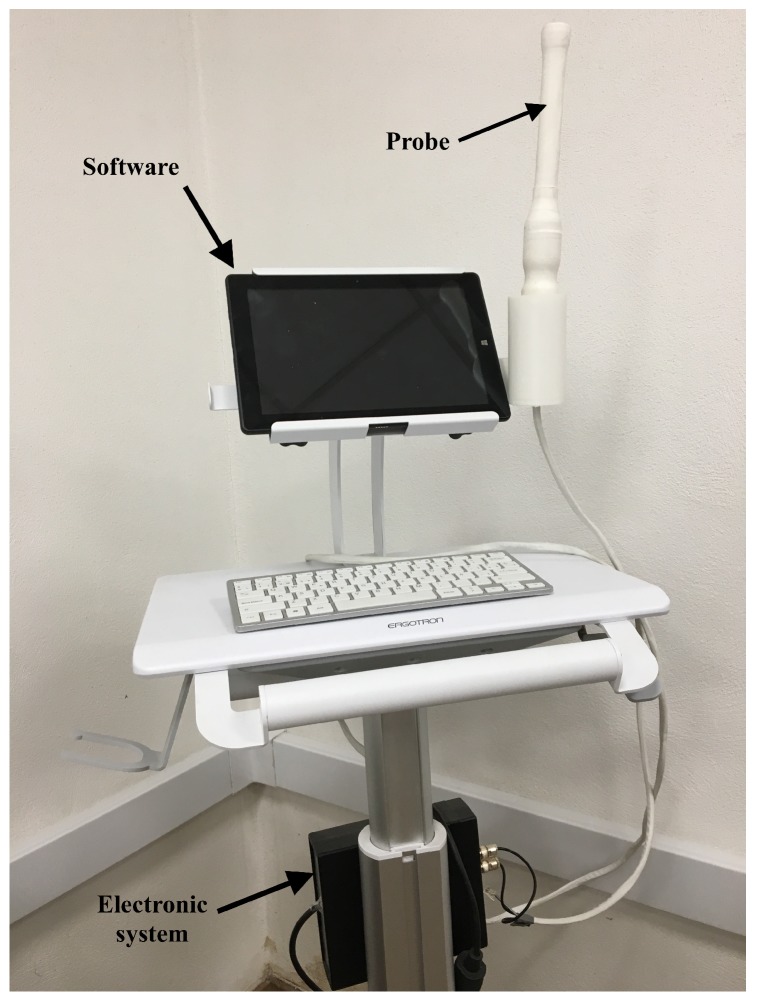
The prototyped TW probe.

Elastography quantification was achieved by the TW probe [26,27,28], which generated waves under safe threshold of energies. The device consisted in three parts: a torsional wave sensor (probe), an electronic system for generating and receiving the signal, and an interface software (Figure 2).

The probe was manufactured in 2017 and was composed of: (1) an electromechanical actuator which deleted electronic cross-talk [23]; (2) a receiver based on two polylactic acid rings where the piezoelectric elements were fitted; and (3) a case to contain the emitter and the receiver. The shear modulus was obtained assuming an elastic and incompressible medium by the following equation,

(1) $$\mu =\rho c_{s}^{2}$$

where ρ is the density of the medium and $c_{s}$ is the torsional wave velocity, which is based on shear wave group velocity.

The excitation signal was a burst composed of a one-cycle frequency *f* ranging from 0.5 to 1.5 kHz with 10× averaging. The frequencies were chosen according to the results obtained in the work carried out by Callejas et al. [23].

**Figure 3:**
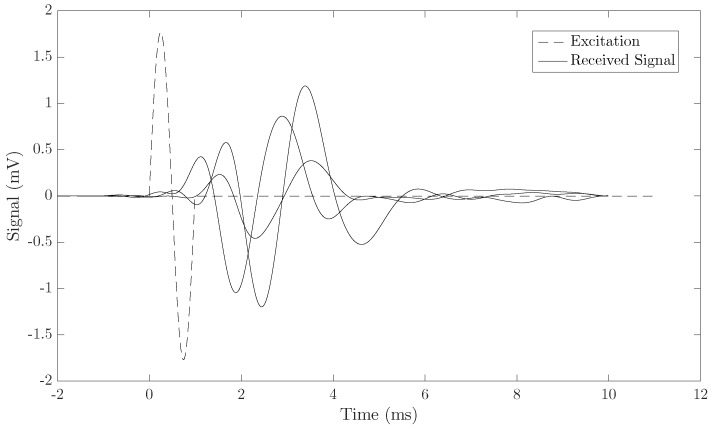
Example of three emitted and received 1 kHz signals.

An example of three different emitted and received signals is shown in Figure 3. The shear wave group velocity calculation algorithm was based on dividing the distance by the torsional wave time-of-flight. The signals were preprocessed by a low-pass filter close to the central frequency of the received signal. The time of flight was computed using three procedures: (1) searching the first time the signal raises 30% above zero; (2) subtracting a quarter of the period (inverse of the received signal central frequency) from the first peak; and (3) subtracting three quarters of the period (inverse of the received signal central frequency) from the second peak. All three methods provided similar estimates of the velocity, as shown in the results.

#### Safety Considerations

A new medical diagnostic equipment needs to follow the specifications described in the Food and Drug Administration (FDA) guidelines [29] for the application in clinical practice. It is necessary that the Torsional Wave technique be safe for humans. There are three parameters that should be evaluated according to the acoustic output in the use of Fetal Imaging and Other (FDA): the mechanical index (MI<1.9), the spatial peak pulse average intensity ($ISPPA<190\;{W/\mathrm{cm}}^{2}$), and the spatial peak temporal average intensity ($ISPTA<94\;{\mathrm{mW}/\mathrm{cm}}^{2}$). The calculation of these parameters was made as follows:

(2) $$MI=PRP/\sqrt{F_{c}}$$

where PRP is the peak rarefractional pressure of the torsional wave in (MPa) and Fc is the center frequency (MHz).

(3) $$ISPPA=P_{0}^{2}/\left(2*\rho *c\right)$$

where $P_{0}$ is the maximal acoustic pressure generated by the electromechanical actuator, ρ is the density of the medium, and *c* is the sound speed in the medium.

(4) ISPTA=ISPPA*Δt/1

where Δt is the excitation pulse duration.

The three parameters were experimentally estimated. The excitation signal used was a low-frequency ultrasonic sine-burst at a central frequency of 1 kHz, consisting of one cycle of 1 ms and 16 Vpp amplitude. This excitation signal was generated by a wave generator (Agilent 33220A, Santa Clara, CA, USA). The response signal was registered using a decibel sensor (YH-610 Environment Multimeter). The signal traveled through a water layer before arriving to the decibel sensor and different distances from 5 cm to 0 cm. To convert the pressure recorded by the decibel sensor into water acoustic pressure, the equation that relates the impedances of the two media (air–water) was used:

(5) $$T=\frac{2*Z_{air}}{{\left(Z_{air}+Z_{water}\right)}^{2}}$$

where *T* is the transmission coefficient and $Z_{air}$ and $Z_{water}$ are the acoustic impedance of the air and water, respectively.

### 2.6. Statistic Analysis

The evolution of cervical stiffness tissue during pregnancy was quantified. Normal distribution of the data was checked for each velocity calculation algorithm by the normal quantile–quantile plot (Q–Q plot) and the Shapiro–Wilk test. The mean values for each velocity calculation procedure were compared to the normal distribution of these values. The coefficient of determination ($R^{2}$) for linear regression analysis was calculated to provide the correlations: (a) between gestational age and cervical velocity ($c_{s}$) using 0.5, 1 and 1.5 kHz torsional waves, for the three velocity calculation algorithms; (b) between gestational age and cervical length; and (c) between stiffness and cervical length. Data were analyzed using the MATLAB (Release 2014b Mathworks, Natick, MA, USA). *T*-test was calculated to estimate *p*-values. A statistically significance for *p* < 0.05 was assumed.

## 3. Results

The experimental results obtained to evaluate the three security parameters according to the Food and Drug Administration guidelines were as follows.

The maximum pressure registered after converting the pressure recorded by the decibel sensor into water acoustic pressure was $3.99\times 10^{-5}$ MPa. The maximal acoustic pressure and the peak rarefractional pressure of the torsional wave in water was $P_{0}=3.99\times 10^{-4}$ bars. The three previous parameters were obtained with the cited experimental conditions:

(6) MI=0.0013<1.9

(7) $$ISPPA=P_{0}^{2}/\left(2*\rho *c\right)=5.3\;{W/\mathrm{cm}}^{2}<190\;{W/\mathrm{cm}}^{2}$$

considering the density of the medium ρ=1000${\mathrm{kg}/m}^{3}$, and the sound speed in the medium 1500 m/s.

(8) $$ISPTA=ISPPA*\Delta t/1=5.3\;{\mathrm{mW}/\mathrm{cm}}^{2}<94\;{\mathrm{mW}/\mathrm{cm}}^{2}$$

**Figure 4:**
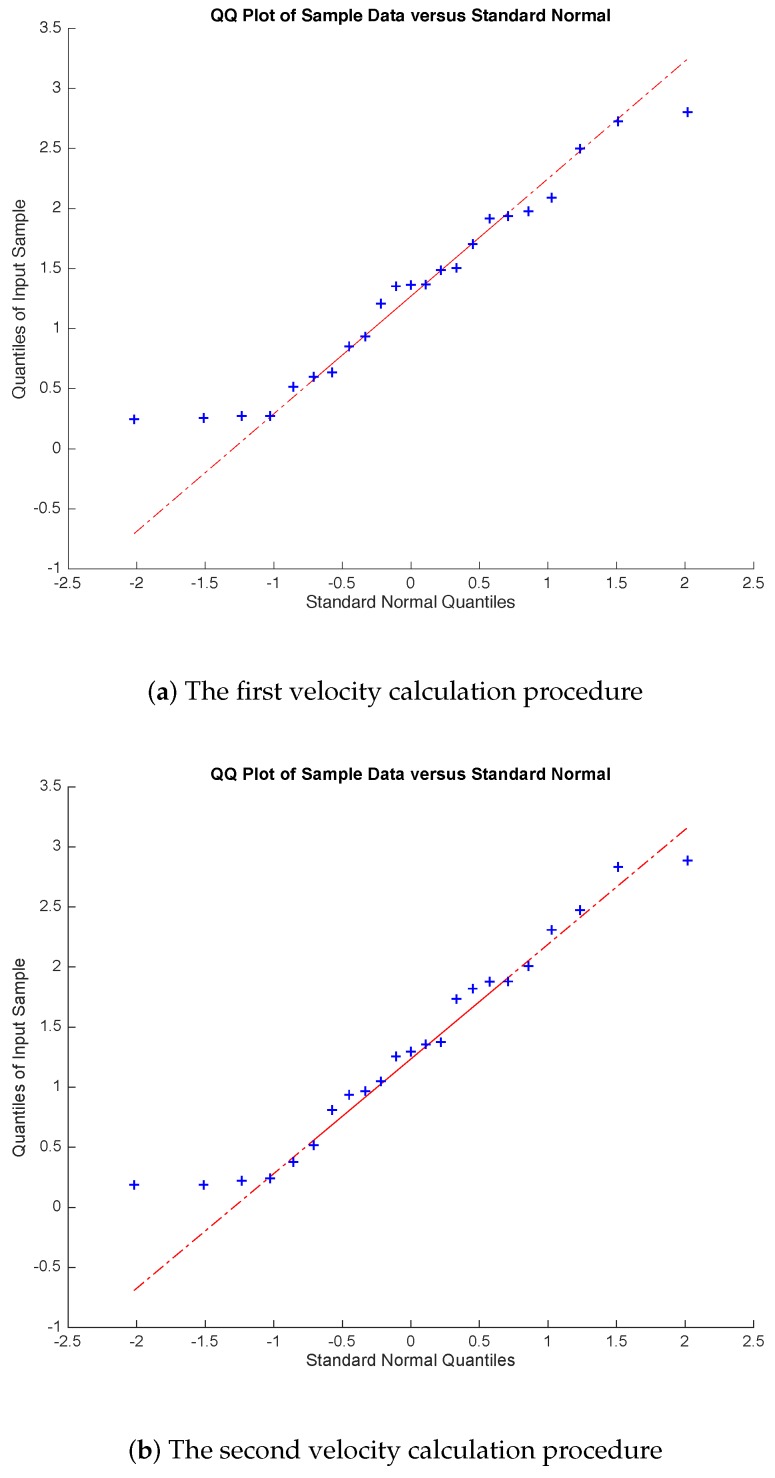

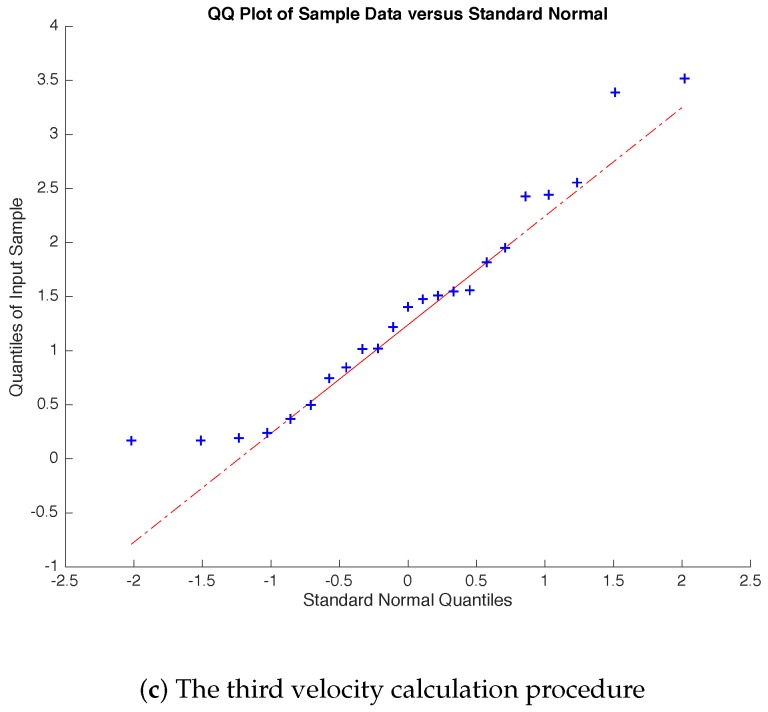
Normal quantile–quantile plots for the three velocity calculation procedures.

A normal distribution for the three velocity calculation procedures was found through Q–Q test (Figure 4) and Shapiro–Wilk test. The obstetric characteristics of the population in the study are shown in Table 1.

**Figure 5:**
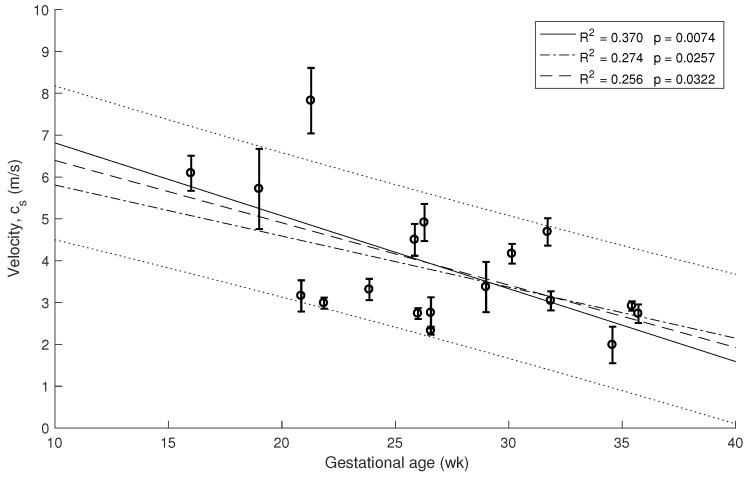
Relationship between cervical stiffness assessed by shear wave speed using 1 kHz waves and gestational age at time of examination.

Three measurements of TW stiffness and cervical length per subject were determined from all women. Box plots of these data observed in each patient were calculated and a linear regression was fitted with 80% confidence intervals (see Figure 5). In this work, the three frequencies were used to study the effect of attenuation on the cervical tissue. The selected frequency configuration was 1 kHz, which was the optimal measure to yield the highest amplitude signals, the best shear wave speed reconstructions and a significant correlation with gestational age. In some measurements, frequencies ≥1.5 kHz, yielded amplitudes of signal similar to the amplitude of noise probably due to attenuation, and consequently anomalous values of the cervical stiffness were obtained. In contrast, the noise masked the amplitude of the signal in some data with frequencies ≤0.5 kHz. All missing data were due to signal noise.

**Figure 6:**
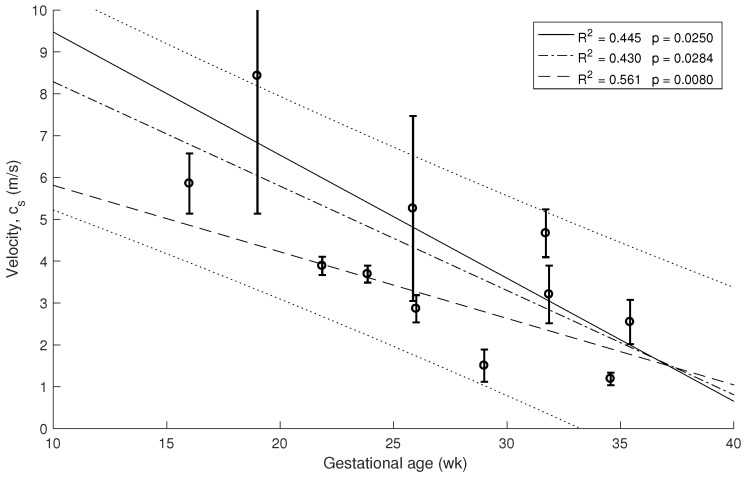
Relationship between cervical stiffness assessed by shear wave speed using 1.5 kHz waves and gestational age at time of examination.

**Figure 7:**
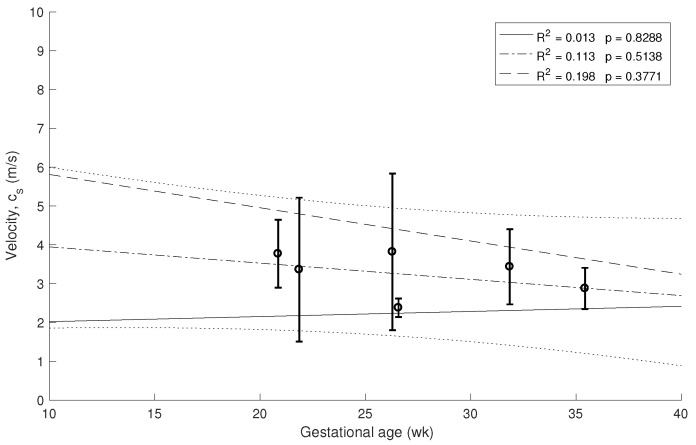
Relationship between cervical stiffness assessed by shear wave speed using 0.5 kHz waves and gestational age at time of examination.

The decrease of the stiffness was computed from the data in Figure 5 using Equation (1). The error bars are estimated from the three velocity estimation algorithms described in the methods. The three overlapping regressions (continuous and dashed lines in Figure 5, Figure 6 and Figure 7) correspond to each velocity estimation algorithms.

**Figure 8:**
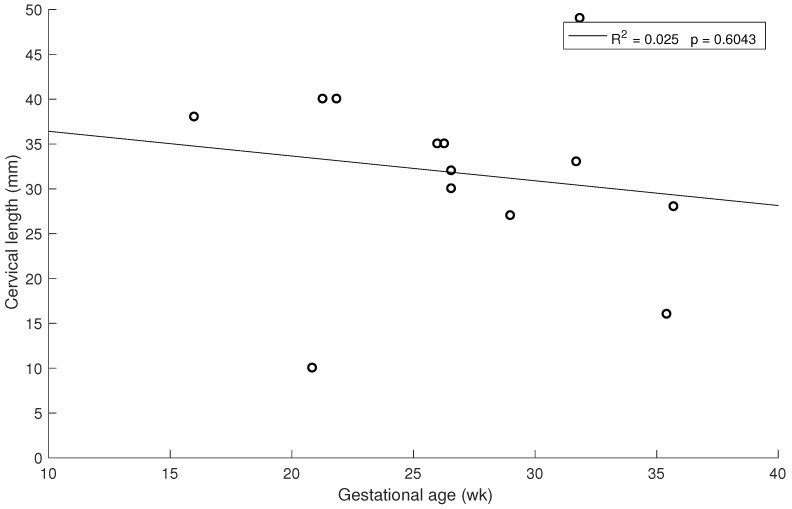
Relationship between cervical length and gestational age at time of examination.

Similar correlations are shown in Figure 6 and Figure 7 for 1.5 kHz and 0.5 kHz, respectively, where some of the measurements were rejected due to noise in the signal. A stronger association between gestational age and cervical stiffness was found ($R^{2}$ = 0.370, p=0.0074, Figure 5) compared to gestational age and cervical length correlation ($R^{2}$ = 0.025, p=0.6043, Figure 8).

**Figure 9:**
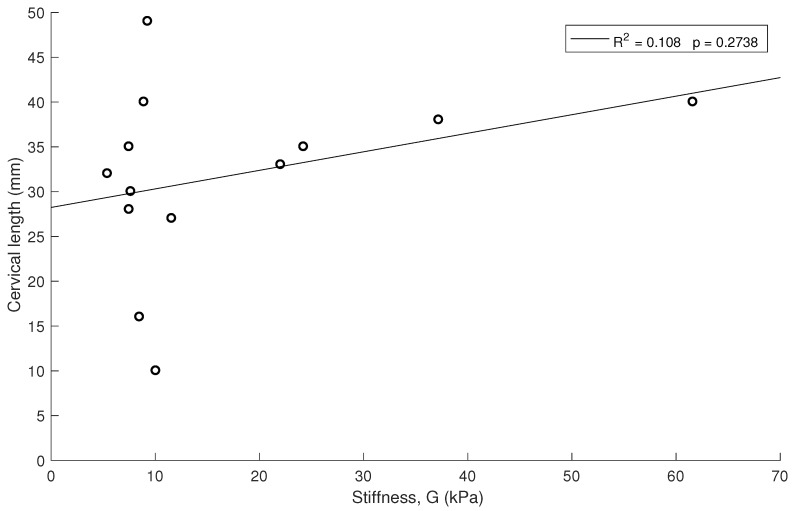
Relationship between cervical stiffness and cervical length.

No high associations ($R^{2}$ < 0.5 for all cases) and no significant correlation (*p* > 0.05) were obtained between stiffness and cervical length (Figure 9).

## 4. Discussion

This study focused on assessing the feasibility of torsional wave technique to quantify the changes in cervical stiffness during pregnancy, which were measured by shear wave speed. The presented results show, for the first time in vivo, the viability of torsional waves to objectively measure cervical elasticity in pregnant women. The observed data therefore support Hypothesis 1 that torsional wave technique has the capacity to quantify cervical stiffness defined by its elastic modulus.

The presented observations also support Hypothesis 2 that shear stiffness decreases during pregnancy. Cervical stiffness was shown to significantly decrease with gestational age, which is compatible with observations by former researchers that assessed cervical ripening by different techniques [8,30,31,32]. A gradual reduction from about 40 kPa at the beginning of pregnancy to close to zero at delivery was obtained in the study carried out by Peralta et al. [30]. A correction due to the difference of range of shear wave frequencies of ARFI was considered, about a higher order of magnitude, which affect the apparent stiffness given the viscoelastic behaviour of cervical tissue. Thus, cervical ripening is directly related to the time to delivery. Correlation between cervical stiffness and gestational age assessed by TW technique showed a higher correlation to gestational aged compared to quantification through shear wave speed (SSI) ($R^{2}=0.37$ vs. $R^{2}=0.29$) [12].

A weaker correlation was found between cervical stiffness and cervical length than with gestational age, which is compatible with previous studies [11,30], using dynamic and quasi-static elastography, respectively, but contrary to observations by Hernandez-Andrade et al. [16], who found that associations between cervical tissue strain and cervical length was higher than with gestational age. This inconsistency feeds a debate, which could be at least partially explained by the inherent limitations of the commercially available quasi-static elastography technologies [14,15,16,17], as this technique provides a qualitative estimation of the cervical stiffness through an indirect measurement.

The experiment results support that TW technique is safe to be used in pregnant women. All the values obtained were far below the thresholds according to the Food and Drug Administration (FDA) guidelines reference parameters in Fetal Imaging and Other. The mechanical index (MI) was 0.0013 (<1.9), the spatial peak pulse average intensity (ISPPA) was $5.3\;{W/\mathrm{cm}}^{2}$ (<$190\;{W/\mathrm{cm}}^{2}$), and the spatial peak temporal average intensity (ISPTA) was $5.3\;{\mathrm{mW}/\mathrm{cm}}^{2}$ (<$94\;{\mathrm{mW}/\mathrm{cm}}^{2}$).

The limitations of this research are linked to the nature of propagation of torsional wave in cervical tissue as well as its complex microarchitecture. Some mechanical hypotheses have been raised in the literature about the hystologic features of the cervix to estimate the shear stiffness elasticity, assuming homogeneous, non viscous, isotropic and semi-infinite medium [20,33,34,35,36]. The equation employed in this study to estimate the cervix stiffness is only based on shear wave group velocity. However, the behavior of cervical tissue is dispersive, that is, the higher are the shear wave frequencies, the higher are the shear waves speeds and, therefore, phase–velocity-based techniques would lead to a direct calculation of shear modulus. The time-of-tflight technique measured the shear wave group velocity, which is dependent on the envelope of the propagating elastic wave.

Finally, due to the exploratory nature of this study about the feasibility of torsional wave technique to assess cervical maturation, a small population of patients was recruited. To extend the validity and reliability of the proposed technology, larger complementary studies are needed. The protocol of measurements by TW technique will be enhanced by applying the optimal contact conditions between the probe and the cervix [37]. We are positive that torsional waves are a tool with potential to objectively diagnose early cervical ripening disorders and preterm birth.

## 5. Conclusions

The presented experimental observations prove that, firstly, cervical stiffness was a valuable predictor variable of gestational age at the moment of evaluation. Secondly, TW technique is a tool that allows quantifying cervical shear stiffness during pregnancy. Finally, this technique is safe to be used in pregnant women.

TW technique might provide clinically relevant data on the cervical ripening in addition to that obtained from digital exploration and standard sonography. Further research is required to assess the TW technique feasibility in obstetric evaluations, such as probabilistic inverse problems based on viscoelastic models for the prediction of preterm delivery and labor induction failure.

## Figures and Tables

**Table 1:** Features of the population in the study.

Characteristics	Value
Total population (N)	18
Gestational age at test (weeks)	26.4 (16 weeks to 35 weeks + 5 days)
Nulliparous (N)	2 (11 %)
Cervical length (mm)	33 (10–49)

## Abbreviations

The following abbreviations are used in this manuscript:

TW	Torsional Wave
WHO	World Health Organization
ARFI	Acoustic Radiation Force Imaging
SSI	Supersonic Shear Imaging
PLA	Polylactic Acid
Internal OS	Internal Orifice of the Cervix
ES	Effect size
